# Numerical Simulation Study on Non-Axisymmetric Die-Less Spinning with a Right-Angle Groove in the Tube

**DOI:** 10.3390/ma18163858

**Published:** 2025-08-18

**Authors:** Xuesong Ren, Zuojun Fan, Zhen Jia, Yongping Shen, Huanzhang You

**Affiliations:** 1 School of Aerospace Engineering, Shenyang Aerospace University, Shenyang 110136, China; 15042172675@163.com (X.R.); 18180848764@163.com (H.Y.); 2 Prosper Metal Forming Corporation, Xi’an 710089, China; shhybm201@163.com

**Keywords:** non-axisymmetric die-less spinning, right-angle groove, finite element simulation

## Abstract

To address the challenges of non-axisymmetric tube spinning, this study employs finite element simulations to validate a novel spinning method for right-angle groove tubes. Three forming schemes with distinct roller path geometries were designed and analyzed using Simufact Forming, with 6063-O aluminum alloy tubes serving as the research material. The simulation results indicate that multi-pass forming (Schemes I and II) significantly enhances wall thickness uniformity compared to single-pass forming (Scheme III). Scheme I exhibits optimal performance due to the minimized equivalent stress in the final forming pass. The maximum stress is concentrated at the groove bottom, leading to wall thinning and springback, while the maximum strain occurs at the roller exit point, where metal accumulation causes local wall thickening. Experimental observations confirm the consistency with the simulation results, validating the model’s reliability. This study deepens the understanding of deformation mechanisms in complex groove forming, highlighting the roller path geometry in controlling stress-strain distribution and final product quality.

## 1. Introduction

As an advanced manufacturing technology characterized by high efficiency, precision, and material utilization, spinning has become indispensable across the aerospace, automotive, defense, and medical industries [1,2,3,4]. Its ability to produce complex shapes with short production cycles makes it particularly suitable for small-batch manufacturing of special-shaped components, driving extensive research on die-less spinning for asymmetric parts [5].

Asymmetric spinning plays a pivotal role in fabricating plates and cylindrical components [6,7,8,9,10]. Wrinkling and cracking in spinning have received much attention. Chen et al. [11] developed a model for flange wrinkling in conventional spinning (combining energy analysis and real-time geometry updates), finding that elastic strain energy oscillations from circumferential shrinkage cause wrinkling. Wilson and Long [12], via FE simulations, found that roller contact induces large residual stress in the flange due to bending moments. Wrinkling in traditional spinning starts when the roller forms a plastic hinge with the blank edge; these moments make wrinkling energy-favorable (shown by reduced elastic strain energy in the model). He et al. [13] used ECCI and APTRS to study cracks in spun Haynes 230 parts, revealing that grain boundary carbide precipitation initiates cracking. For asymmetric cylindrical parts, Wilson et al. [14] pioneered offset tube production by adjusting the relative position between the tube axis and rotation axis, thus expanding geometric design possibilities. Arai and Gondo [15] introduced a multi-pass spinning technique to form slanted or curved tubes, enhancing process flexibility for non-axisymmetric profiles. Jia et al. [16] further demonstrated the formation of saddle grooves in tube mid-sections using conventional rollers through optimized roller path derivation, overcoming limitations of traditional symmetric spinning.

The increasing demand for complex geometries has spurred the integration of finite element simulation into spinning research. Huang et al. [17] utilized Abaqus/Explicit to numerically investigate high-temperature neck spinning, analyzing how the friction coefficient, roller speed, and tip radius influence forming quality. Zhao et al. [18] proposed a hybrid spinning-flaring method for uniform-thickness curved workpieces, elucidating deformation mechanisms and failure modes such as compression buckling in transition zones. Xia et al. [19] focused on hot spinning of thin-walled magnesium alloy tubes with internal ribs, revealing defect formation mechanisms such as underfilling and back depression in rib structures. Complementary studies have deepened the understanding of spinning mechanics. Gondo et al. [20] characterized thickness, texture, and crystal orientation changes in aluminum tube spinning, linking microstructural evolution to process parameters. Xu et al. [21,22] identified non-periodic stress distributions caused by uneven roller contact, proposing a three-roller staggered configuration to balance mandrel loads and improve operational stability. Their follow-up work on titanium alloy spinning revealed that only the McClintock model accurately predicts crack reduction under moderate thinning rates, highlighting model specificity in material-dependent processes. Roy et al. [23] validated axisymmetric finite element models’ limitations in predicting non-axisymmetric outcomes, while Xiao et al. [24] optimized roller paths for flanged cylindrical components, determining a 21° maximum flange inclination through parametric analysis. Xia et al. [25,26,27] pioneered non-axisymmetric die-less spinning on tube ends and validated the method via 3D finite element modeling. Subsequently, Jia et al. [17,28] extended this research to the mid-section of the tube, further exploring non-axisymmetric die-less spinning in central regions.

Special-shaped tubular structures exhibit considerable potential application value, and there currently exist typical forms such as saddle-shaped asymmetric structures. However, the special shape of the tube with straight features in the middle has not been thoroughly investigated. This study focused on right-angle groove structures with distinct linear features as the analytical object. Since the stress–strain characteristics of the metal flow field are difficult to obtain directly through experimental means, the finite element simulation method is thus proposed for this research. Through systematic comparative analysis with the experimental results, the reliability of the finite element simulation results is verified. On this basis, by utilizing the simulation results, the distribution characteristics of the stress–strain field under three working conditions are analyzed, and the stress concentration areas and weak parts in the forming process are clarified, which provides a theoretical basis for subsequent actual production.

## 2. Establishment of the Finite Element Model

### 2.1. Roller Path Design

**Figure 1:**
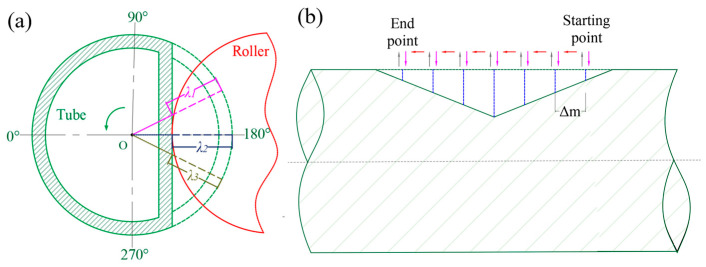
Schematic representation of roller path. (**a**) Radial direction. (**b**) Axial direction.

To produce a tube with a right-angle groove, the axial and radial movements of the roller are strategically designed. In the radial direction, the roller periodically approaches the tube and retracts according to a specified feed depth. Axially, after completing each radial pass, the roller shifts to the next position at a specified feed rate to prepare for the subsequent radial pass. As depicted in Figure 1a, the feed depth of the roller is dependent on the angular position of the workpiece, denoted by *λ*_1_*, λ*_2_, and *λ*_3_. When the workpiece rotates 180°, the roller reaches its maximum feed depth, corresponding to the depth *λ*_2_ (represented by the blue line). This depth signifies the maximum depth of the current channel. Concurrently, as illustrated in Figure 1b, once the radial feeding is completed, the roller initiates axial movement. After advancing Δm, the axial motion ceases. When the tube rotates to the designated position, the roller resumes radial movement. This cyclic process is repeated until the forming is completed.

**Figure 2:**
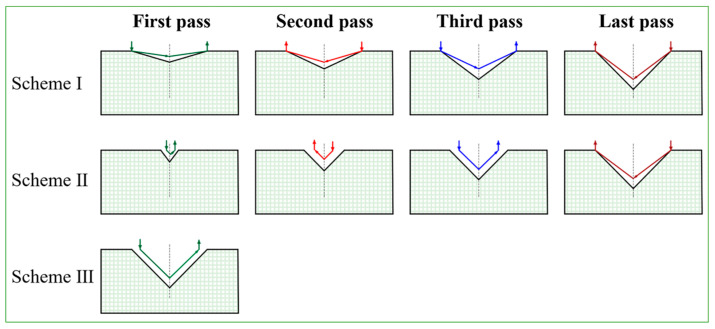
Representations of the axial roller paths for three schemes.

To optimize the forming process, three distinct forming schemes were designed, comprising two multi-pass forming schemes and one single-pass forming scheme. Scheme I maintains a constant forming width for each pass while gradually increasing the forming depth; Scheme II keeps the forming angle unchanged across passes but progressively increases both the forming width and depth; Scheme III achieves direct forming with the maximum width and depth in a single pass. This systematic design of three schemes investigates the effects of process parameters (width, depth, and wall thickness) on forming quality, providing a theoretical foundation for selecting optimal process routes in practical applications. The specific details of each scheme are illustrated in Figure 2. The final forming depth for all spinning schemes is fixed at 9 mm.

**Figure 3:**
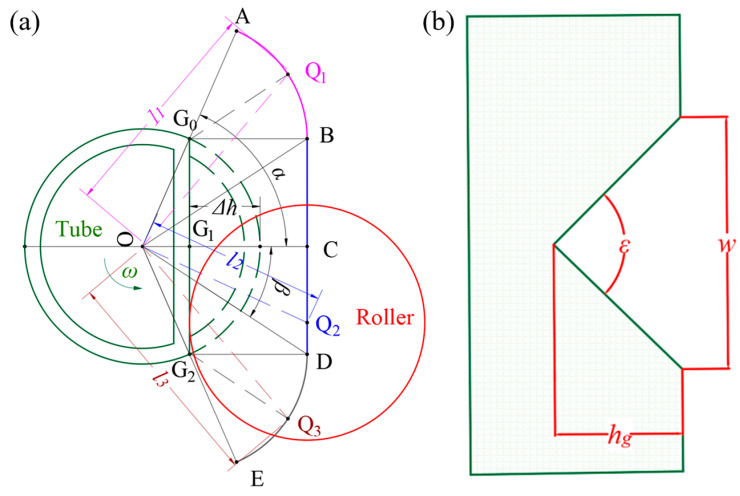
The geometric relationship of the roller path. (**a**) Radial direction. (**b**) Axial direction.

To form a tube with a right-angle groove at its midsection into the target shape, a reasonable roller path is critical. The tube is mounted on a shaft for rotation, and the roller combines axial and radial reciprocating movements; in the radial direction, the roller moves around the tube in the non-deformation region and contacts the tube to initiate deformation. The deformation area path is divided into three stages, with the specific geometric relationship of the radial path illustrated in Figure 3a.

Stage 1 involves the roller center moving from initial point A to boundary point B; Stage 2 involves the roller center moving from B to D at the maximum feed depth, with C (the midpoint of line segment BD) corresponding to the maximum feed depth position; Stage 3 involves the roller center moving from D to the final point E. The critical contact points include G_0_ (the first contact point in the current pass), G_1_ (the contact point at maximum feed depth), and G_2_ (the last contact point before deformation ends), where Q denotes the roller center position and O denotes the tube center position. To determine the rotation angle of each stage, let the angular velocity be Ω and the time be *t*, where *R* = roller radius, *r* = tube radius, and Δ*h* = maximum reduction in the previous pass. In triangle G_0_OG_1_, ∠G_0_OG_1_ is denoted as *α*, and its cosine value is given by

(1) $$\alpha =arccos\left(\frac{r-\Delta h}{r}\right)$$

In triangle BOC, ∠BOC is denoted as *β*, and its cosine value is expressed by

(2) $$\beta =\arctan(\frac{\sqrt{r^{2}-{\left(r-\Delta h\right)}^{2}}}{r+R-\Delta h})$$

The angular ranges of the three stages are as follows:

(3) $$\left\{\begin{array}{l} \pi -\alpha <\Omega t<\pi -\beta \\ \pi -\beta <\Omega t<\pi +\beta \\ \pi +\beta <\Omega t<\pi +\alpha \end{array}\right.$$

Let the position of the center during Stage 1 be denoted as Q_1_. Consider triangle G_0_OQ_1_, where the length OQ_1_ is *l*_1_, and ∠G_0_OQ_1_ is *γ*_1_. Then, *γ*_1_ can be expressed as

(4) $$\gamma_{1}=\Omega t-\pi +\alpha$$

Then, according to the cosine theorem, *l*_1_ can be expressed as

(5) $$l_{1}=r\left[\cos\gamma_{1}+\sqrt{\cos^{2}\gamma_{1}-{\left(R/r\right)}^{2}+1}\right]$$

Let the position of the center during Stage 1 be denoted as Q_2_. Consider triangle COQ_2_, where the length OQ_2_ is *l*_2_, and ∠COQ_2_ is *γ*_2_. Then, *γ*_2_ can be expressed as

(6) $$\gamma_{2}=\left|\pi -\Omega t\right|$$

Then, according to the cosine theorem, *l*_2_ can be expressed as

(7) $$l_{2}=(R+r-\Delta h)/\cos\gamma_{2}$$

Let the position of the center during Stage 3 be denoted as Q_3_. Consider triangle G_2_OQ_3_, where the length OQ_3_ is *l*_3_, and ∠G_2_OQ_3_ is *γ*_3_. Then, *γ*_3_ can be expressed as

(8) $$\gamma_{3}=\pi +\alpha -\Omega t$$

Then, according to the cosine theorem, *l*_3_ can be expressed as

(9) $$l_{3}=r\left[\cos\gamma_{3}+\sqrt{\cos^{2}\gamma_{3}-{\left(R/r\right)}^{2}+1}\right]$$

By combining Equations (1)–(10), the complete feed rate *δ* can be derived as

(10) $$\delta =R+r-\left\{\begin{array}{l} l_{1},\pi -\alpha <\Omega t<\pi -\beta \\ l_{2},\pi -\beta <\Omega t<\pi +\beta \\ l_{3},\pi +\beta <\Omega t<\pi +\alpha \end{array}\right.$$

As illustrated in Figure 3b, the axial target shape is similar to an isosceles triangle, with a base width of *w*, a depth of *h_g_*, and a vertex angle of *ε*, which satisfies the following relationship:

(11) $$\tan\frac{\varepsilon }{2}=\frac{w/2}{h_{g}}$$

Let the axial feed rate be *z*(*t*), the workpiece rotational speed be *f_rot_*, and the axial feed rate be *f_ax_*; then, *z*(*t*) can be expressed as

(12) $$z(t)=f_{ax}f_{rot}t$$

**Figure 4:**
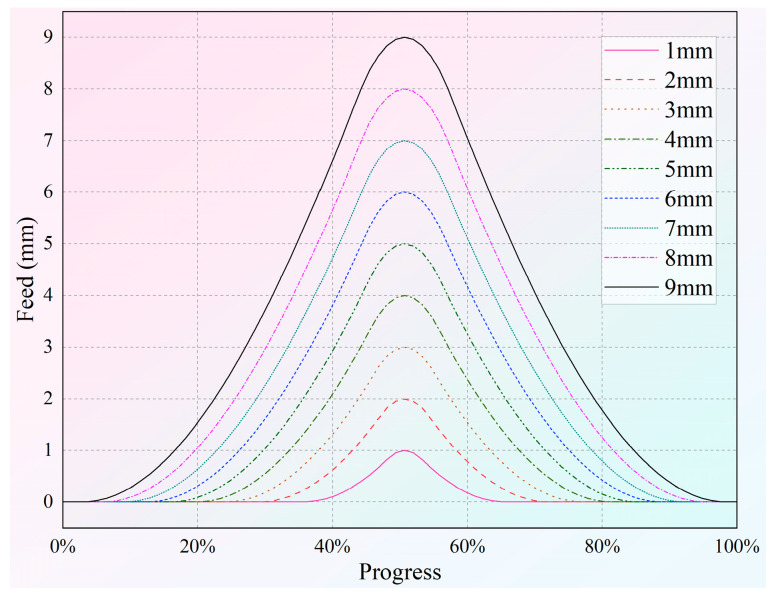
The radial feed of the roller.

Drawing on Equations (1)–(12), the coordinated control of the roller’s axial and radial feed can be effectively achieved. Thus, a comprehensive experimental procedure can be established to meet the specified shape requirements. The radial feed of the roller for forming depths ranging from 1 to 9 mm is detailed in Figure 4. The axial feed can be designed according to three schemes.

### 2.2. Material Parameters

**Figure 5:**
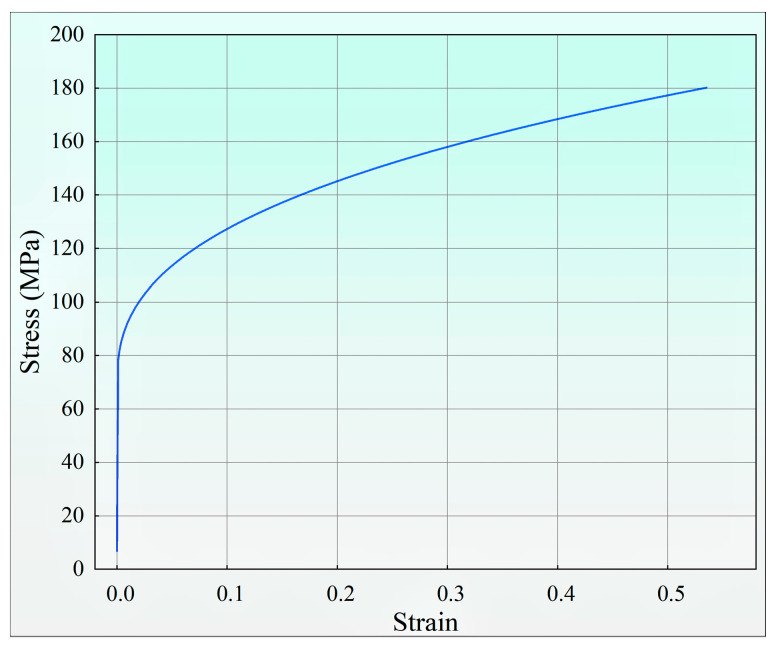
Stress–strain curve of 6063-O aluminum alloy.

The tube material selected was 6063-O aluminum alloy. Its detailed chemical composition and mechanical property parameters are listed in Table 1 and Table 2 [16,29,30], respectively. The corresponding stress–strain curves are illustrated in Figure 5. The constitutive equation of 6063-O aluminum alloy at room temperature is described by a power-hardening model, expressed as

(13) $$\sigma =\sigma_{s}+K\varepsilon^{n}$$

In this model, the flow stress σ is defined as the instantaneous stress required to sustain plastic deformation. The plastic strain *ε* is referenced to the yield point, with *ε* = 0 corresponding to the onset of yielding. Here, σ_s_ denotes the yield strength of the 6063-O alloy, *K* is the strength coefficient, and n is the strain-hardening index. These parameters were determined by fitting experimental stress–strain data, yielding σ_s_ = 68 MPa, K ≈ 142.35 MPa, and n ≈ 0.38. For regions without plastic deformation (where ε = 0), the material obeys Hooke’s law (*σ* = E*ε*), and the stress remains below the yield strength.

### 2.3. Settings of the Finite Element Model

In this study, computational models were established using the Simufact Forming 16.0 finite element simulation platform. Three-dimensional geometric models were constructed for key components, including the forming roller, tube, spindle, and tailstock. With the exception of the tube, other components exhibit extremely small and negligible deformation, so they are treated as rigid bodies. Specifically, the mandrel and roller are modeled as rigid bodies due to their significantly higher hardness (45 steel, hardness > 180 HB) compared to the 6063-O tube (annealed, hardness ~55 HB), resulting in negligible tool deformation under actual forming forces (maximum ~0.4 kN). However, minor effects persist—such as a slight overestimation of local contact pressure—though these are negligible for predicting overall trends. Notably, for softer tooling (carbon steel) or extreme forces exceeding the tools’ yield limits, the rigid-body assumption may introduce larger errors, though such scenarios lie beyond the scope of this study.

The tube was fixed via two-point fixation using a spindle and a hydraulically driven tailstock; one end was inserted into the spindle, and the other end was secured by the tailstock’s conical tip, which extended into the tube and applied a pressure of 2–3 MPa. This ensured coaxial rotation of the tube with the spindle (runout < 0.03 mm) and prevented axial slippage. To simplify the model, the spindle and tailstock were merged into a single model, with corresponding forces applied to simulate their functional roles. Mineral oil containing anti-wear additives was uniformly sprayed onto the tube’s outer surface and the roller (flow rate: 5 mL/min), maintaining a Coulomb friction coefficient of 0.12 ± 0.02 (with 0.12 adopted in simulations). The roller and spindle components were calibrated, ensuring dimensional accuracy within ±0.02 mm, and the perpendicularity deviation between the roller axis and the tube axis was controlled to <0.1°. The tube’s wall thickness and outer diameter were measured using a digital micrometer and vernier caliper. In the simulation, a table-driven approach was adopted to ensure the accuracy of the roller’s axial and radial movements. This means that all movements are time-synchronized, with each time point corresponding to a specific position of the roller.

**Figure 6:**
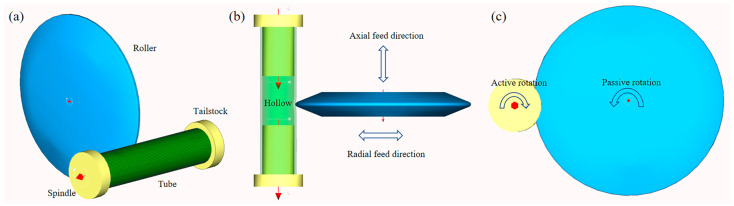
The 3D geometric models of the spinning device. (**a**) Axonometric view. (**b**) Top view. (**c**) Left view.

The radius of the roller is 75 mm. Material properties can be directly simulated by inputting the corresponding data into the software. As depicted in Figure 6, the tube has the following dimensional specifications: an overall length of 120 mm, an outer diameter of 30 mm, and a wall thickness of 2 mm. The tube was firmly mounted between the spindle and tailstock mechanism, with both ends equipped with 40 mm long axle insertions to guarantee precise concentric alignment between the tube, spindle, and tailstock components. The target forming section of the tube remained unconstrained within the central hollow area. The contact surface of the roller featured a small nose radius of 2 mm. The experiment was conducted at room temperature, so the temperature was set to 25 degrees Celsius. Material properties can be simulated by inputting the data from Table 1 and Table 2, along with Equation (13), into the software.

The finite element model was constructed using 3D hexahedral elements (Sheetmesh) with reduced integration, which is well suited for handling large plastic deformation and mitigating mesh distortion. To balance computational efficiency and simulation accuracy, a uniform meshing strategy was adopted with a consistent mesh size throughout the entire model. The consistent mesh size was set to 0.5 mm, determined by the geometric characteristics of the tube (wall thickness 2 mm) and the right-angle groove (minimum radius 2 mm). This size ensures that the deformation zone (e.g., contact area between the roller and tube) is discretized into at least 4 elements along the thickness direction, which is sufficient to resolve local strain gradients while avoiding excessive computational load. This ensures sufficient resolution to capture local strain gradients in deformation zones (contact areas between the tube and roller) while maintaining a manageable computational load. A mesh convergence study was performed for the 6063-O aluminum alloy tubes with a 2 mm wall thickness, focusing on the number of element layers through the thickness (1, 2, and 3 layers). Considering the significant local strain gradients in deformation zones, the three-element layer configuration, despite increasing the computation time by 15% compared to the two-element layer, more accurately captured the stress–strain distribution, especially in stress-concentrated regions such as the groove bottom. Quantitative comparison showed that the one-element layer underestimated the maximum equivalent stress at the groove bottom by 12% compared to the three-element layer, while the two-element layer exhibited a 5% deviation. The three-element layer, despite increasing the computation time from 8 h to 9.2 h (on a 16-core workstation), achieved a convergence error of <2% relative to a finer four-element layer, confirming its optimality. Thus, the three-element layer mesh was selected as optimal for subsequent simulations.

The nonlinear finite element solver MSC.Mar 11.13 was employed for the simulations, with the key solver parameters configured as follows: the timestep was dynamically adjusted within the range of 1 × 10^−6^ to 1 × 10^−5^ s, scaled by the smallest element size in the model to ensure numerical stability. The scaling followed the Courant–Friedrichs–Lewy (CFL) condition with a safety factor of 0.3, i.e., timestep = 0.3 × (smallest element size/maximum material flow velocity). For the 0.5 mm element size, the maximum flow velocity (≈0.1 m/s during peak deformation) yielded the calculated timestep range, ensuring numerical stability in high-strain regions. Convergence criteria were defined as a force residual threshold of <1 × 10^−5^ N and a displacement tolerance of <1 × 10^−6^ m, ensuring sufficient accuracy for capturing deformation characteristics. The force residual threshold corresponds to 0.1% of the maximum forming force (≈0.4 kN) measured in experiments, while the displacement tolerance is 0.5% of the minimum wall thickness change (0.2 mm) observed, ensuring that the simulated results match the experimental accuracy.

## 3. Simulation Results and Verification

**Figure 7:**
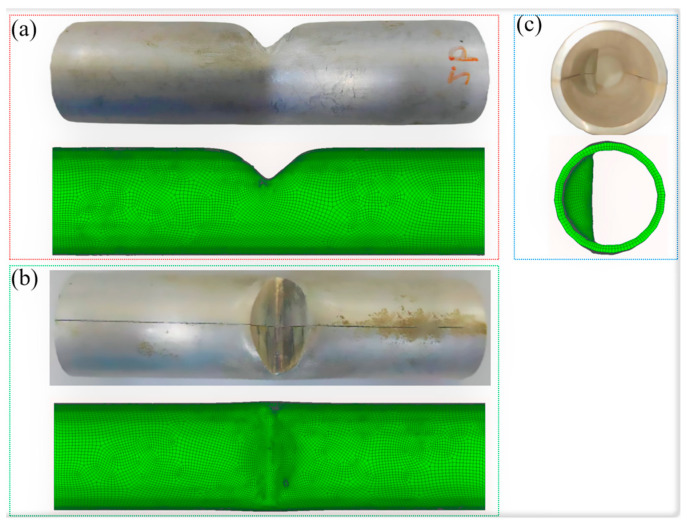
Comparison of the experimental and simulation results. (**a**) Main view. (**b**) Top view. (**c**) Left view.

To validate the reliability of the simulation results, this study took Scheme II as the comparison object. A comparison of macroscopic appearances between the simulations and experiments is shown in Figure 7. The shapes from both the simulations and experiments are highly consistent.

**Figure 8:**
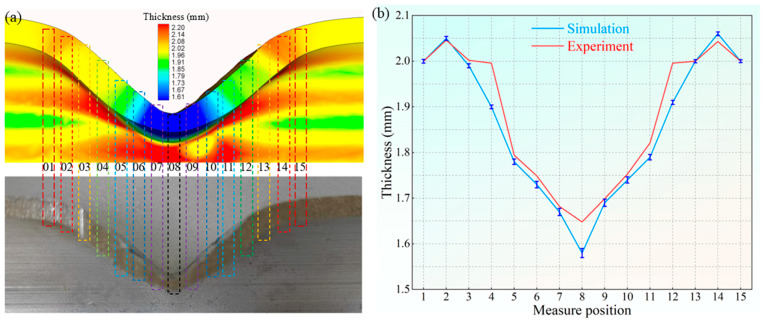
The wall thickness distribution. (**a**) Axial sections of the experimental and simulation results. (**b**) The wall thickness result.

To accurately measure the wall thickness, the tube is cut along the axial direction (as indicated by the tangent line in Figure 7). To further validate the simulation reliability, the simulated wall thickness was compared with the experimental data. Figure 8 illustrates the wall thickness distribution at 15 positions along the axial section. The maximum wall thickness difference between the simulation and experiment at position 4 is 4.8%. The experimental and simulated wall thickness results are in close agreement.

Table 3 presents the forming depth, width, and angle from both the simulation and experiments. All experiments were performed in triplicate, with mean values calculated from repeated measurements. The maximum relative error rates are 12.1% for the forming depth, 7.8% for the forming width, and 5.1% for the forming angle. The measured groove dimensions in both the simulation and experiments were recorded after complete unloading to account for postforming elastic recovery, which emerges as the dominant factor for depth discrepancies. For 6063-O aluminum, the material exhibits significant springback after spinning; the simulations predict a depth reduction of ≈0.21–0.33 mm due to elastic recovery (from the maximum roller feed to the final dimension), while the experimental measurements show an additional ≈ 1–1.1 mm reduction. This total springback of 1.2–1.4 mm explains why both results fall below the nominal 9 mm target, with the 12.1% maximum discrepancy arising from the slight overestimation of plastic deformation in simulations, and the springback was not fully simulated. Friction variations further contribute to angle deviations (up to 5.1%). The simulations adopted a constant Coulomb friction coefficient (0.12) across the contact interface, whereas experimental lubrication showed localized unevenness. This non-uniform friction altered the plastic flow direction near the apex, causing the experimental angle to be 2–4.5° steeper than the simulated values.

Similar errors also occurred in [28], and they were within an acceptable range. There are acceptable differences between the simulation results and the experimental results, which confirm the accuracy.

## 4. Discussion

This section explores the forming mechanism of right-angle groove spinning, involving a thorough analysis of the forming process, equivalent stress, and strain distribution. Based on the simulation results, these aspects will be systematically expounded and discussed in the following sections.

### 4.1. Analysis of the Forming Process

**Figure 9:**
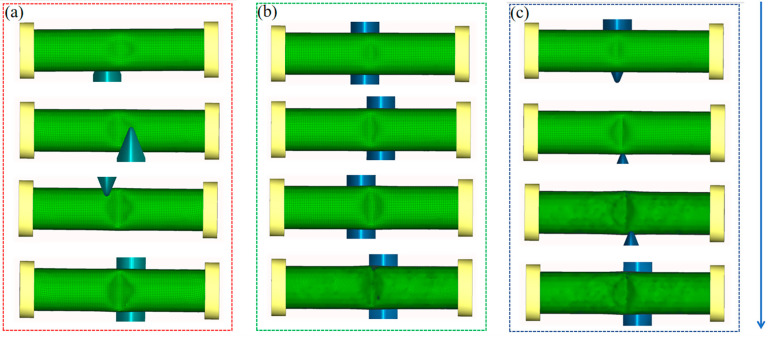
Deformation process of right-angle groove spinning. (**a**) Scheme I. (**b**) Scheme II. (**c**) Scheme III.

The spinning process is illustrated in Figure 9. Scheme III is single-pass forming. As the roller makes periodic contact with the tube, a local straight slope gradually develops in the middle of the tube until reaching the maximum forming depth of 9 mm. Initially, one slope of the right-angle groove is formed, and the process continues until the other slope is completed, finally forming a fully developed right-angle groove. The forming processes of Schemes I and II are analogous; through multi-pass spinning, small local grooves are generated at the end of each pass, progressively accumulating until a complete 9 mm deep right-angle groove is achieved. This multi-pass deformation mechanism indicates that the spinning device can be effectively modeled with roller paths in a 3D finite element model, capturing the incremental formation of complex geometries via roller–tube interactions.

**Figure 10:**
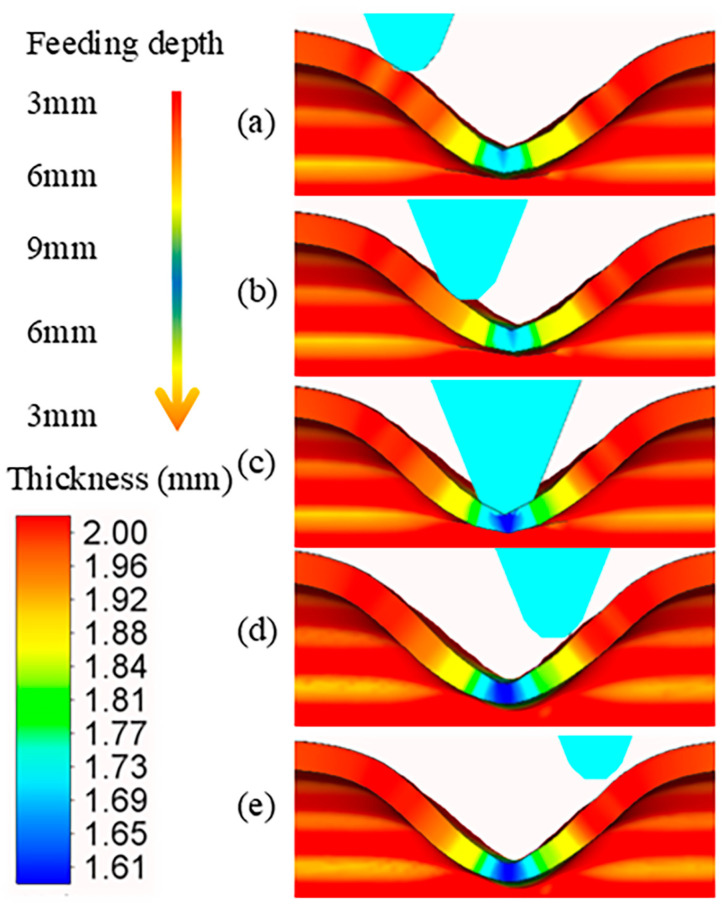
Wall thickness variation in the axial section. (**a**) 3 mm (lower slope). (**b**) 6 mm (lower slope). (**c**) 9 mm (maximum). (**d**) 6 mm (upper slope). (**e**) 3 mm (upper slope).

In order to analyze the wall thickness distribution characteristics in right-angle groove spinning, a key issue arises from non-axisymmetric deformation. Taking Scheme II as an example, each pass forms a partial right-angle groove profile. Focusing on the fourth pass, Figure 10 illustrates the wall thickness measurements at five axial–tangential positions. Notably, the wall thicknesses of the two straight slopes exhibit symmetry at the same forming depth, while a slight thickening occurs in the transition zone between the deformed and non-deformed regions. This phenomenon is attributed to the resistance from the non-deformed material at the end of each forming pass, which restricts metal flow and causes local accumulation. With the increase in forming depth, wall thinning becomes more significant. When the depth is 3 mm, no obvious thickness change can be observed. As the depth reaches 6 mm, the thinning amount exceeds 0.15 mm. At the final depth of 9 mm, the maximum thinning at the groove bottom approaches 0.42 mm. A larger feed depth leads to higher forming forces, leading to more significant wall thickness thinning.

**Figure 11:**
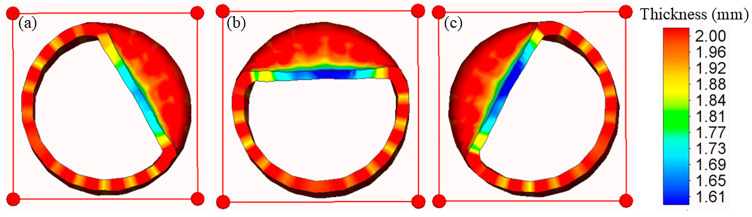
Wall thickness variation in the radial section. (**a**) Before forming. (**b**) During forming. (**c**) After forming.

For the tangential cross-section at a forming depth of 9 mm (as depicted in Figure 11), thinning occurs across the deformed zone, with the most substantial reduction being observed in the central area. The preforming central thinning was approximately 0.25 mm, which increased to nearly 0.4 mm during the forming process, accompanied by minimal post-forming variation. This suggests that wall thinning mainly occurs during the stage of maximum axial feed, when the roller exerts the highest radial force, thereby driving intensive metal flow along the axial direction. The wall thickness change is also evident between the roller entry and exit sides; the entry side exhibits more pronounced thinning, whereas the exit side accumulates more material due to successive roller passes, which results in higher resistance and localized thickening.

In summary, non-axisymmetric die-less spinning of right-angle grooves results in a non-uniform wall thickness distribution, characterized by maximum thinning at the right-angle groove vertex and partial thickening at the deformation boundary.

### 4.2. The Stress Distribution

**Figure 12:**
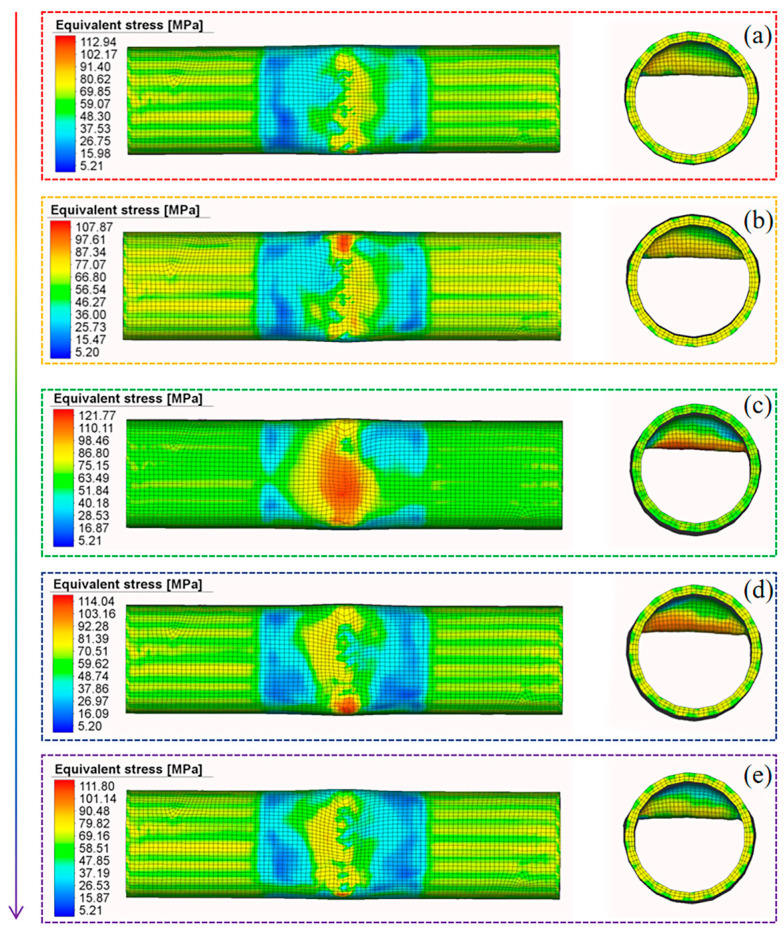
The equivalent stress distribution. (**a**) Pre-forming. (**b**) Stage 1. (**c**) Stage 2. (**d**) Stage 3. (**e**) Final forming.

Figure 12 illustrates the equivalent stress distributions at various stages of the fourth pass with a forming depth of 9 mm for Scheme II. Since the deformation trajectory of the roller is divided into three stages, the stress distribution of each stage is observed as follows: the forming process is divided into the following stages: pre-forming (preparatory setup before deformation), stage 1 (initiation of deformation), stage 2 (application of maximum feed depth (9mm)), stage 3 (transformation nearing completion), and final forming (completion of the process).

The analysis reveals that during pre-forming, stress concentrates on one slope of the groove (the recently processed area), reflecting equivalent stress from previous passes. In Stage 1, stress is concentrated in the roller’s contact area, and the feed depth is minimal at this stage. The process then enters Stage 2; as the forming depth increases, the stress magnitude gradually increases, resulting from the cumulative plastic deformation caused by the roller’s incremental penetration. When the process enters Stage 3 and the feed depth decreases, the stress decreases accordingly. After the forming is completed, the roller finally separates from the tube. Notably, the maximum stress consistently remains at the bottom of the right-angle groove throughout the entire process. The high equivalent stress in this area is prone to causing springback, thereby affecting forming accuracy.

Different spinning schemes not only directly determine the groove’s shape and size but also affect wall thickness and stress–strain distributions, which, in turn, influence the mechanical properties of the spinning component.

**Figure 13:**
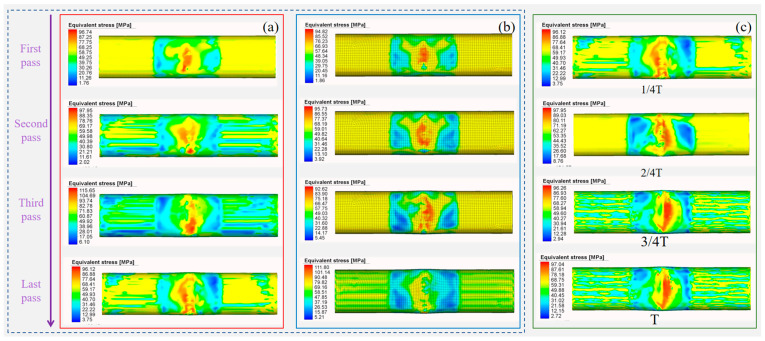
Equivalent stresses of the three schemes. (**a**) Scheme I. (**b**) Scheme II. (**c**) Scheme III.

The maximum stress occurs at the maximum forming depth. By comparing the equivalent stress after the maximum forming depth in each pass of Scheme I and Scheme II, the forming effects of different schemes can be evaluated. As Scheme III involves single-pass forming, four uniformly selected positions based on the forming sequence are used to observe the forming effect, and T represents the working condition progress, as shown in Figure 13. Single-pass forming in Scheme III leads to unidirectional metal flow, eventually causing stress to concentrate on one side of the groove. Although the target shape can be achieved, excessive wall thickness variation results in an inferior forming effect.

Similar phenomena occur in Scheme I and Scheme II. However, in multi-pass forming, the incremental metal flow in each pass and the reversed flow direction between passes weaken this effect. In both schemes, equivalent stress concentrates at the bottom of the right-angle groove, with little difference between the schemes. Scheme I exhibits slightly higher equivalent stress in the first three passes than Scheme II but lower equivalent stress in the last pass. Notably, the last pass has the most significant impact on the forming process, as previously processed areas undergo reprocessing. Consequently, Scheme I demonstrates slightly better forming performance than Scheme II.

**Figure 14:**
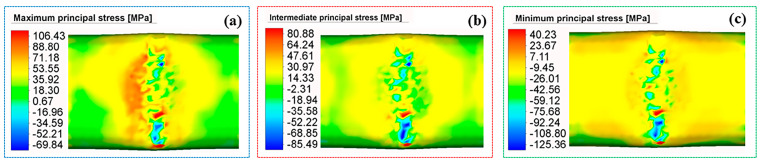
The multidirectional stress distribution in the deformation zone. (**a**) Maximum principal stress. (**b**) Intermediate principal stress. (**c**) Minimum principal stress.

The multi-directional stress distribution in the post-forming deformed region is presented in Figure 14. For the maximum principal stress (Figure 14a), tension dominates, with a peak value of 106.43 MPa. The high-stress area concentrates on the slope of the roller exit side, forming a band-shaped distribution extending along the roller’s pressing direction. This area aligns with the equivalent stress concentration zone at the groove, with both reflecting the core deformation principle of the spinning process. The intermediate principal stress (Figure 14b) exhibits a distinct mixed tension-compression state; a tensile peak of 80.88 MPa and a compressive valley of −85.49 MPa coexist spatially. This duality mirrors the non-uniform circumferential deformation constraints—the stronger constraint on one side of the groove induces compressive stress, while the weaker constraint on the opposite side triggers tension. For the minimum principal stress (Figure 14c), compression is dominant, with a valley value of −125.36 MPa. Its high-magnitude compressive stress zone closely coincides with the bottom of the groove, corresponding to the radial extrusion deformation of the tube wall (where the roller presses the wall, causing radial thickness reduction and compressive stress concentration). The distribution pattern of the three principal stresses intuitively reveals the mechanical field characteristics of “local forced deformation” in spinning: stress concentration with tension-compression coupling in the core contact zone and a gradient decay of stress magnitude toward the non-contact transition zone. These features align well with the experimental observations of deformation uniformity and stress-induced springback.

### 4.3. The Strain Distribution

**Figure 15:**
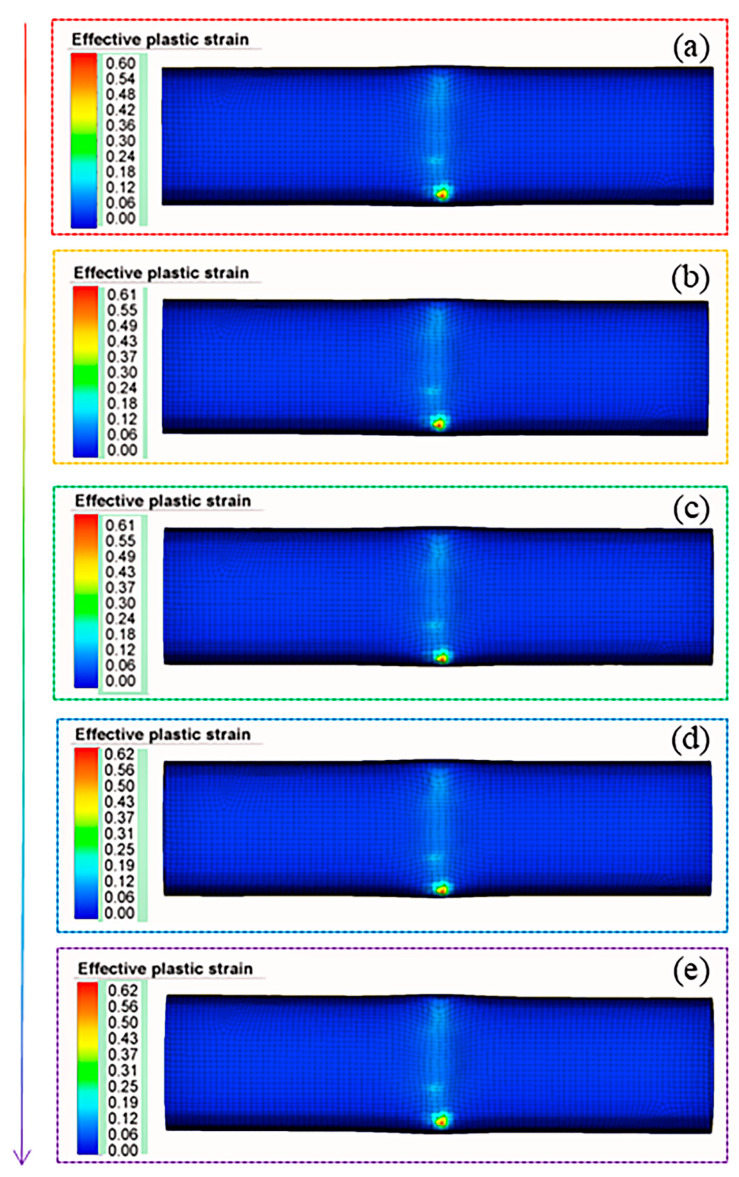
The equivalent strain distribution. (**a**) Pre-forming. (**b**) Stage 1. (**c**) Stage 2. (**d**) Stage 3. (**e**) Final forming.

Similarly to Figure 12, Figure 15 depicts the equivalent strain distribution of the fourth pass with a forming depth of 9 mm for Scheme II. Slight strain variation is observed before and after forming, while the maximum strain occurs in the roller exit area. This is attributed to metal accumulation caused by the roller at this location, where the roller’s action leads to increased wall thickness and significant deformation.

Notably, the strain at the bottom of the right-angle groove is significantly higher than in other regions and is directly correlated with the progressive wall thinning as the forming depth increases. The underlying mechanism can be summarized as follows: at the roller exit (specifically at the interface between the deformed and non-deformed zones), tangential stress exceeds that in other regions, causing localized metal accumulation. Here, the metal flow is predominantly tangential, with a minor axial movement component. This phenomenon becomes more pronounced with a deeper forming depth.

**Figure 16:**
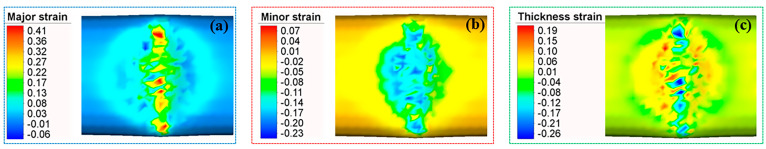
The multidirectional strain distribution in the deformation zone. (**a**) Major strain. (**b**) Minor strain. (**c**) Thickness strain.

To further analyze the deformation, the post-molding deformation area was selected as the observation target. As shown in Figure 16, the major strain, minor strain, and thickness strain in the spinning deformation zone exhibit distinct distribution characteristics: the major strain, dominated by tensile deformation (peak value: 0.41) and concentrated in the bottom of the groove, reflects dominant axial constraints; the minor strain, with a mixed positive and negative distribution (negative peak: −0.23), reveals uneven circumferential constraints; the thickness strain, centered on radial thinning (peak: −0.26) and strongly coupled with the major strain, aligns with the constant volume assumption in plastic deformation, consistent with the aforementioned content.

## 5. Conclusions

This study focuses on non-axisymmetric die-less spinning of right-angle grooves in the middle of a tube, verifying the proposed method’s credibility via 3D finite element simulation. The core contents are as follows.

(1) The macroscopic shape and wall thickness variations from simulations and experiments are largely consistent, validating the model’s accuracy and demonstrating the feasibility of the proposed forming method.
(2) Single-pass forming exhibits severe stress concentration, uneven wall thickness, and unidirectional metal flow, leading to low forming accuracy. Multi-pass forming mitigates stress concentration through staged deformation. Scheme I outperforms Scheme II because of the lower equivalent stress in the last pass and more uniform wall thickness distribution.
(3) The deformation zone experiences overall wall thickness thinning. The maximum stress localizes at the bottom of the right-angle groove, increasing with the forming depth. Springback causes the actual depth to be slightly less than the ideal value (9 mm). The strain at the groove bottom is higher than in other regions due to the more pronounced wall thickness thinning, while the strain in the roller exit region is significantly elevated by localized thickening due to metal accumulation.

## 6. Prospects

In future research, the geometric parameters of the roller and roller trajectories can be further optimized to quantitatively investigate their effects on the forming accuracy of right-angle grooves. Additionally, the research scope can be expanded to include high-strength alloys such as titanium alloys and stainless steel, focusing on the stress–strain behavior of these alloys during forming. The integration of artificial intelligence algorithms to optimize multi-pass forming paths is also promising, as it can improve control over wall thickness uniformity. Further improving the accuracy of numerical simulations remains an important research objective. Finally, the development of novel methods for forming more complex geometries will be explored to expand the applicability of this spinning technique.

## Figures and Tables

**Table 1:** Chemical composition of 6063-O aluminum alloy.

Element	Content Range (%)	Typical Value (%)
Aluminum (Al)	Balance	—
Silicon (Si)	0.20–0.60	0.40
Magnesium (Mg)	0.45–0.90	0.65
Iron (Fe)	≤0.35	0.25
Copper (Cu)	≤0.10	—
Manganese (Mn)	≤0.10	—
Zinc (Zn)	≤0.10	—
Titanium (Ti)	≤0.10	—
Other Impurities	Single ≤ 0.05, Total ≤ 0.15	—

**Table 2:** Mechanical properties (typical values at room temperature) of 6063-O aluminum alloy.

Property	Test Standard [31]	6063-O Aluminum Alloy
Tensile Strength (σ_b_)	ASTM B221	≤180.2 MPa
Yield Strength (σ_s_)	ASTM B221	≤68 MPa
Relative Elongation (δ_5_)	ASTM B221	≤56%
Hardness (HB)	ASTM E18	≤50 HB
Elastic Modulus (E)	—	68–70 GPa
Poisson’s Ratio (ν)	—	0.33

**Table 3:** The forming depth, width, and angle.

	Scheme	Depth (mm)	Width (mm)	Angle (°)
	I	7.64	18	95.34
Experiment	II	7.60	16	92.93
	III	7.74	17	94.36
	I	8.73	17.9	91.43
Simulation	II	8.67	17.4	90.19
	III	8.79	17.5	89.73

## Data Availability

The original contributions presented in this study are included in the article. Further inquiries can be directed to the corresponding authors.
